# A wave of support? A natural experiment on how the COVID-19 pandemic affected the popularity of a basic income

**DOI:** 10.1057/s41269-022-00260-9

**Published:** 2022-10-05

**Authors:** Arno Van Hootegem, Tijs Laenen

**Affiliations:** 1 Centre for Sociological Research, KU Leuven, Parkstraat 45, 3000 Louvain, Belgium; 2 Tilburg School of Social and Behavioral Sciences, Tilburg University, Tilburg, The Netherlands

**Keywords:** Public preferences, Basic income, COVID-19 pandemic, Polarization, Natural experiment

## Abstract

Although a basic income (BI) has already been widely debated, the COVID-19 crisis further intensified the discussion about this periodic cash payment that is unconditionally delivered to all. However, it remains unclear whether the crisis spurred a wave of public support for its introduction. To investigate this, we aim to answer two research questions: (1) How did support for a BI evolve in reaction to the COVID-19 crisis? and (2) To what extent did the evolution in support differ across regional contexts and social groups with varying levels of deprivation? We rely on a natural experiment by analysing data from the Belgian National Elections Study that was collected both before and during the pandemic. The results indicate an increase in support for a BI due to the pandemic, although it seems short lived and not necessarily specific to a BI. Importantly, however, the increase in popularity is only observable in the high-unemployment French-speaking region of Belgium and among relatively deprived groups.

**Supplementary Information:**

The online version contains supplementary material available at 10.1057/s41269-022-00260-9.

## Introduction

Even before the outbreak of the global pandemic that brought life to a standstill, the idea of a basic income (BI) was gaining momentum among a wide audience of academics, policymakers and politicians. Yet in response to the COVID-19 crisis, the appeal of a BI that is granted to all citizens on an individual basis without means testing or a work requirement became even greater. Among others, the United Nations Development Program advised introducing a temporary BI to protect vulnerable citizens in developing countries (George and Ortiz-Juarez 2020). However, the crisis spurred a boost in BI experiments and petitions to pressure governments to introduce this income benefit, including in Europe. Especially in light of the lurking economic recession and surging unemployment rates, a BI is considered to be an attractive alternative to existing welfare arrangements. Not only can it function as a more encompassing social safety net, but a BI is also argued to serve as a stimulus for the economy by encouraging consumption and offering a more efficient benefit system (Ståhl and MacEachen 2021). While it should, hence, be clear that a BI is considered a viable policy option to deal with challenges arising from the COVID-19 crisis, it remains unclear whether this goes hand in hand with a wave of support for a BI among the general public. Such public support is nevertheless crucial, in view of the political feasibility of taking a BI from a promising policy idea to actual policy implementation.

Intuitively, one might expect support for BI to increase as part of a larger movement of solidarity that has resonated across Europe. For instance, both local initiatives that aimed to help those in need and large citizen participation programmes sprang up in the first months after the outbreak of the pandemic, as part of an apparent shift towards a more solidaristic culture. Because in its ideal–typical form a BI encompasses a universal and unconditional type of welfare provision that may effectively reduce poverty in society and provide more encompassing social protection (Van Parijs and Vanderborght 2017), this potential upsurge in sympathy for the fate of the worst off could result in increased popularity of a BI. However, this positive evolution in solidarity in general, and support for a BI in particular, mostly relies on indirect evidence and is far from certain. Indeed, the broader literature on the impact of heightened risk exposure in times of crisis indicates that people can have a wide variety of reactions to increasing uncertainty. Although people may become more positive towards welfare provision (Cappelen et al. 2021; Nettle et al. 2021), it has also been argued that they may become less altruistic (Brito Vieira et al. 2017; Jensen and Naumann 2016) when risks intensify. Moreover, it is very possible that the evolution in opinion is not homogeneous across social groups, as different socio-economic strata are disproportionally affected by crises and may, hence, not change their opinion in tandem (Patel et al. 2020).

In light of this, the current study tries to answer two specific research questions: (1) *How did support for the introduction of a BI evolve in reaction to the COVID-19 crisis?;* and (2) *To what extent did the impact of the COVID-19 crisis on support for a BI differ across social groups and contexts with varying levels of deprivation?* To answer these questions, we adopt a natural experiment that allows us to approximate the average causal treatment effect of being exposed to the COVID-19 crisis (Dunning 2012; Jensen and Naumann 2016). In particular, by comparing the attitudes of citizens who participated in a large probability-based survey before the outbreak of the pandemic with the opinions of citizens interviewed after the introduction of the first government lockdown, we are able to more directly estimate the effect of the crisis on support for a BI. Most previous studies have simply asked respondents to give their retrospective opinions before the pandemic or have primed them with a number of COVID-related questions before formulating their attitudes of interest (WeMove Europe 2020; Nettle et al. 2021), thereby only yielding a (potentially inaccurate) proxy of the effect of the crisis. Moreover, our approach assesses whether the COVID-19 crisis and its associated increased risk exposure mobilized citizens to become more sympathetic towards a BI in the short term. This is in contrast to previous research that has usually compared post-crisis attitudes with a baseline of several years before (Brito Vieira et al. 2017; Gonthier 2017; Reeskens et al. 2021), thereby potentially overestimating stability in welfare preferences. In addition, we contribute to the literature on how crises affect welfare opinions generally, as well as on the impact of the COVID-19 crisis specifically, by evaluating how increased risk exposure influences the attitudes of different types of social groups. Instead of only focusing on differences between *individuals* with alternative levels of precarity, as previous research has mostly done (Brito Vieira et al. 2017; Cappelen et al. 2021; Gonthier 2017), we also examine (dis)similarities across *groups* that experience varying levels of relative deprivation and across *regional contexts*. In this way, we uncover whether polarization in the impact of the COVID-19 crisis has mainly occurred in relation to individuals, social groups or regional contexts. To realize this, a descriptive overview and several regression analyses are provided on the basis of data from the Belgian National Elections Study, 2019–2020.

## Theoretical perspectives on the impact of the COVID-19 crisis on support for a BI

### COVID-19 and public support for a BI

One could ask why it is particularly interesting to study support for a BI in light of the COVID-19 crisis, instead of examining more general attitudes towards redistribution. For one thing, specific preferences about more strongly disputed issues—including the desirability of a BI—are generally expected to be more volatile and can change to a larger extent in reaction to disrupting events (Reeskens et al. 2021). Public opinion concerning a BI may, hence, be more suitable to use as a dependent variable to study how increased risk exposure affects welfare-related preferences. Yet more importantly, in its ideal–typical form a BI is a radically new proposal that deviates substantially from existing welfare arrangements. Its unconditional and universal nature not only distinguishes it from many types of institutionalized welfare provision (Van Parijs and Vanderborght 2017), but is also the exact reason why it could be considered as a more viable policy option during a crisis. In times of increasing inactivity, declining working hours and substantial income losses, existing benefit schemes that rely for instance on work-related history may be seen as insufficient to meet the growing need for social protection. In this sense, a universal and unconditional benefit that is not tied to work contributions and covers all inhabitants may have a natural appeal during a prolonged period of hardship.

Most prior research nevertheless reveals little about how public support for BI evolves over time and is silent about the extent to which it is affected by crisis situations. There are two notable exceptions. The first is the study by Nettle et al. (2021) (see also an analysis of their data in Weisstanner 2022), who aimed to investigate whether the COVID-19 pandemic increased support for a BI among (non-representative) samples of the UK and US populations. Their main conclusion is that support for a BI is substantially higher in times of a pandemic than in normal times. Importantly, this increase in popularity persisted (albeit less so) even six months after the start of the pandemic and proved specific to a BI. That is, it was especially a BI that grew in popularity, compared with a targeted and conditional welfare scheme. This finding was interpreted as a confirmation of the assumption that COVID-19 increased support for a BI, rather than overall support for any type of welfare provision. The second survey, by WeMove Europe (2020), compares support for the introduction of an EU-wide BI with and without explicitly referring to the pandemic and its economic impact. Based on results from six European countries, the conclusion reached is that support for the introduction of a BI is slightly higher when people are actively reminded of the COVID-19 crisis.

There is, however, one very important caveat to the data of both Nettle et al. (2021) and the WeMove Europe Survey (2020): instead of comparing responses before and after the occurrence of the pandemic, respondents were either just primed with a reference to COVID-19 or merely asked to retrospectively rate how they supported a BI before the pandemic, as if it had never happened. This was then compared with the support they showed for the policy in times of the pandemic. Although Nettle et al. (2021) recognize that a longitudinal, experimental design with a pre-pandemic and a peri-pandemic measurement would have been better, they seem to assume that their sub-optimal study design actually underestimates the opinion shift in favour of a BI: ‘If respondents’ normal-times responses were contaminated by their current high level of support due to the ongoing pandemic, then the true opinion shift they have undergone in the last six months or so may be larger than our study implies’ (Nettle et al. 2021, p. 11).

With the help of a unique natural experiment, our study aims to scrutinize the extent to which a shift in public support for a BI has actually taken place, and if so, the extent to which it is persistent and specific to a BI. However, in contrast to the intuitive hypothesis of Nettle et al. (2021) that COVID-19 has produced an across-the-board increase in support for a BI, our theoretical framework suggests that: (1) the pandemic might equally have led to a decrease in such support, and (2) the over-time trend in support will be likely to vary across individuals, social groups and regional contexts experiencing different levels of structural and incidental, crisis-induced precariousness.

### An increase or decrease in support?

Two competing theories on how attitudes change in light of crisis situations can be found in the broader literature on how exogenous shocks affect welfare-related preferences. First, the so-called ‘government protection thesis’ assumes that needs for welfare provision increase during times of crisis, as people become more exposed to a number of risks, such as unemployment or income loss. This increased need for government protection heightens self-interest in public provision and can in turn lead to more positive attitudes towards the welfare state (Blekesaune 2007; Jæger 2013; Sachweh 2018). However, welfare attitudes research has traditionally also recognized a competing hypothesis, labelled here as the ‘cost awareness hypothesis’. According to this view, heightened risk exposure can induce self-regarding reasoning and can weaken concerns for the well-being of the disadvantaged in society, thereby eroding public support for the welfare state (Brito Vieira et al. 2017; Durr 1993; Sihvo and Uusitalo 1995). In times of hardship, citizens may become more aware of increasing costs and limited resources, thereby being more hesitant to support extensive government provision (Jensen and Naumann 2016).

It is difficult to decide which of the two theoretical perspectives will be most appropriate to describe the evolution of support for a BI in light of the COVID-19 crisis. On the one hand, based on the potential of a BI to address some of the key challenges arising from the pandemic, an increase in the popularity of a BI could be expected among the general public. The COVID-19 crisis increased both economic and health risks, in turn leading to a greater need for a social safety net, thereby potentially increasing support for a BI. This seems to be corroborated by some empirical studies; for example, Cappelen et al. (2021) find stronger solidarity in the United States when people are reminded to think about the COVID-19 crisis, as do Nettle et al. (2021) for the case of a BI (see above). On the other hand, the general public may be concerned with rising social expenditure during the pandemic, and hence, become less altruistic, in turn making them sceptical about implementing even broader income support measures. Indeed, Daniele et al. (2021) demonstrate that when individuals are primed with questions on the pandemic and its consequences, they are less inclined to support welfare spending financed by taxation, while Asaria et al. (2021) reveal that individuals experiencing economic or health shocks during the pandemic are less inequality averse. Because both the theory and the empirical evidence point in different directions, we formulate two contrasting hypotheses:

**H1a** Support for a BI will be higher during the COVID-19 pandemic than before (government protection hypothesis)

**H1b** Support for a BI will be lower during the COVID-19 pandemic than before (cost awareness hypothesis)

The specific nature of the COVID-19 crisis and the peculiarity of a BI nevertheless raise additional questions that are not fully covered by existing theory on the general evolution of welfare attitudes in light of crises. First of all, the pandemic naturally occurred in waves, and the temporary character of the crisis was emphasized from its start. In tandem, the economic and social consequences of the crisis closely followed the rate of infections, with businesses as well as social life closing down and opening up again based on the severity of health risks. As a result, one could question the extent to which this has implications for the persistence of potential shifts in public opinion, and whether support for welfare provision may be short term and in response to fluctuating risk exposure. To test this, we also study how support for a BI fluctuated across the months of the pandemic and in line with the rate of COVID-19 related hospitalizations.

Moreover, a BI is a particular type of welfare proposal, and its legitimacy cannot be blindly equated with support for other forms of social policies. Indeed, people often differentiate between their support for a BI and other redistributive schemes (Weisstanner 2022). In addition, a BI is intrinsically also fundamentally multidimensional, as it can vary in terms of many different design characteristics, such as universality, conditionality and integration within existing welfare schemes (De Wispelaere and Stirton 2004). This implies that support for a BI is best measured as support for different types of BI that vary on these design dimensions (Laenen et al. 2022; Rincón 2021; Stadelmann-Steffen and Dermont 2020). Prior studies on the impact of COVID-19 have nevertheless only analysed how it influences support for the ideal–typical version of a BI, which is both universal and unconditional (Nettle et al. 2021). Accordingly, we also study what types of BI became more or less popular during the pandemic, in order to see whether it was truly the uniquely universal and unconditional character of a BI that drove changes in support.

### Parallel publics or increasing polarization?

Even when establishing the general direction in which support for a BI has evolved, the question remains whether this trend is universal across groups with varying levels of material deprivation. Some people were arguably more strongly affected than others by the COVID-19 pandemic and its consequences, potentially leading to diverging reactions. With regard to the general evolution of support for a BI, existing literature wavers between two contrasting theoretical expectations of how different groups react to increased risk exposure. On the one hand, when assuming that people reason more from a self-interested perspective, different experiences of the crisis could lead groups to alter their opinions on welfare provision in various ways (Brito Vieira et al. 2017; Forma 2002). This so-called ‘polarization thesis’ predicts that groups that are disproportionally affected by a crisis demand more government protection, whereas the middle and upper classes may turn against generous welfare provision because they fear the costs will rise exponentially. On the other hand, the ‘parallel publics’ hypothesis predicts that external shocks will have equivalent repercussions across different groups, as they are exposed to very similar narratives and information during a crisis (Gonthier 2017; Page and Shapiro 1992). Moreover, even if various groups are differentially impacted, they can still have the same national interest at heart when formulating their opinions and they may reason beyond their own self-interest out of sociotropic concerns (Page and Shapiro 1992).

The COVID-19 pandemic certainly affected groups with varying levels of deprivation differently, which could potentially offer fertile breeding ground for increased polarization in opinions concerning the implementation of a BI. Individuals with lower socio-economic status were not only disproportionally exposed to COVID-19 and more unequal health outcomes, but their unstable working conditions and financial insecurity were also exacerbated (Patel et al. 2020). At the same time, there was a strong feeling that we were ‘all in the same boat’, as the pandemic also forced relatively well-off workers and the self-employed into (temporary) unemployment, and it became clear that everyone was at risk of being infected with the virus. The empirical evidence from prior studies examining sub-group differences in welfare-related attitudes during the pandemic is conflicting. On the one hand, some studies seem to confirm the polarization hypothesis. For example, Ares et al. (2021) show that heightened ideological polarization occurred regarding welfare state capacity, efficiency and political trust in the wake of the pandemic. On the other hand, there are also studies that appear to confirm the parallel public hypothesis. For example, Cappelen et al. (2021) demonstrate that there was a universal trend towards greater solidarity in the wake of the COVID-19 crisis, and Weisstanner (2022) shows that priming people with pandemic-related questions had a relatively uniform impact across social groups on their support for a BI. As with the general trend in support, it is, therefore, difficult to determine a priori which of the two theoretical hypotheses is most suitable to predict how support for a BI evolved among different groups after the COVID-19 crisis. Accordingly, two contrasting hypotheses are again formulated:

**H2a** The evolution of support for a BI will follow diverging trends for different social groups and regional contexts with varying levels of deprivation (polarization hypothesis)

**H2b** The evolution of support for a BI will follow a similar trend for different social groups and regional contexts with varying levels of deprivation (parallel publics’ hypothesis)

To investigate which theoretical prediction holds true, we compare the impact of the COVID-19 crisis across groups and contexts that differ in their relative degree of deprivation. In contrast to previous studies, we do not focus on a single operationalization of deprivation, but instead examine three dimensions that correspond to the individual, group and contextual level. First, we compare individuals with a (perceived) low income and those with a high one, as it has been shown that income and financial hardship are important predictors of support for a BI (Choi 2021; Lee 2018). However, in addition to this individual (or household) measure of hardship, we focus on the role of group-relative deprivation, which plays a substantial role in forming people’s opinions about the welfare state (Van Hootegem et al. 2021). Group-relative deprivation encompasses the feeling that one’s ingroup is disadvantaged compared with other societal groups, resulting in frustration and anger towards outgroups and the social system (Smith et al. 2012). Rather than precarious positions per se, group-level experiences could be decisive in the convergence or polarization of support for a BI in the wake of the COVID-19 crisis. Last, in addition to these individual and group-level indicators, we also compare the impact of the pandemic across regional contexts. In particular, as our data were collected in Belgium, a comparison between Flanders and Francophone Belgium is made. Francophone Belgium has been more deprived than Flanders in recent decades, with worse socio-economic performance and higher unemployment rates (Working Group Social Impact Corona Crisis 2021), which could translate into a different effect of the COVID-19 crisis. For example, in 2020 the unemployment rate was more than twice as high in Francophone Belgium (Wallonia = 7.4%; Brussels = 12.4%) than in the Flemish region (3.5%) (Statbel 2020). By focusing on individual experiences of hardship as well as on shared group sentiments and regional contexts, we are able to offer a more robust appraisal of the parallel publics and polarization hypotheses.

## Data and methods

### Research design

We analyse data from the Belgian National Elections Study of 2019–2020, a large probability-based survey. The National Register functioned as the sampling frame, and citizens 18 years and older entitled to vote were interviewed. Because voting is compulsory in Belgium, this means that our sample is taken from the entire adult population. The COVID-19 pandemic interrupted the data collection and resulted into two periods during which respondents were interviewed: the first group of respondents (*N* = 955) was interviewed between December 2019 and March 2020 (pre-pandemic), and the second group (*N* = 704) between June 2020 and November 2020 (peri-pandemic). While the first group of respondents was interviewed using only Computer Assisted Personal Interviewing (CAPI), the second group was interviewed using a combination of CAPI, video calls and Computer Assisted Web Interviewing (CAWI). Respondents with missing values are omitted from the analyses, and weights according to age, gender and education are applied.

As the data collection was split in two, coinciding with periods before and after the Coronavirus outbreak in Belgium, we can treat data collection as a type of natural experiment. By comparing support for a BI across the two groups, we can approximate the average causal treatment effect of the crisis. However, two important conditions are connected to this claim: (1) there is an *as-if* randomization to the control and treatment group, and (2) attitudes would remain stable in the hypothetical absence of the treatment (Dunning 2012; Jensen and Naumann 2016). The assignment to both groups *as-if* it was random can be checked by determining whether there are systematic differences in the types of respondents interviewed in the two samples. Table A1 in the Online Appendix shows the descriptive statistics for respondent characteristics in the two groups, as well as the results of a logistic regression model that predicts membership of the COVID sample relative to the pre-COVID sample. Some important differences are apparent, as French-speaking, younger and lower-educated respondents are more likely to be part of the treatment group. This indicates that assignment is not fully random. However, the potential bias from these imbalances (Dunning 2012) is corrected for by controlling for these characteristics in the regression models (Jensen and Naumann 2016). The second prerequisite, which states that attitudes should be stable in the absence of treatment, is hypothetical and, hence, impossible to test. A series of t tests nevertheless highlight that no significant fluctuations in support for a BI occurred in the months before the outbreak of the pandemic, while there is a significant difference between support for a BI before and after the start of the pandemic. This strengthens our belief that stability in preferences would have been observed without the occurrence of the COVID-19 crisis.

### Indicators

The dependent variable is support for the introduction of a BI, measured using a vignette experiment that randomly varied the characteristics of the BI under consideration. The introduction text of the vignette was formulated as follows: ‘In some countries there is a debate about whether to introduce a BI. In a moment, I will ask you to what extent you are in favour or against this BI. First, I will give you some more information about what we understand by a BI’. After this introduction, the characteristics of the specific BI were varied according to five dimensions, with each having two to four levels: universality, uniformity, conditionality, integration and accumulation. The sixth and last characteristic of the particular BI is the same for all respondents and mentions that the BI is paid for by taxes, thus, emphasizing that its implementation is funded by public resources. Table A2 in the Online Appendix displays the wordings of all the different levels of each of these dimensions. However, as we are not primarily interested in these different experimental conditions to evaluate the impact of the COVID-19 crisis, our analyses mostly control for the type of BI that people are presented with. The variation in specific BI types—some of which deviate significantly from the ideal–typical definition of a cash payment made to all without means testing and work requirements—nevertheless allows us to examine the extent to which COVID-19 increased (or decreased) support for a universal and unconditional BI, rather than support for welfare provision in general. The responses to the BI vignette were on an 11-point scale (0 = strongly against; 10 = strongly in favour).

In addition to a dummy that indicates whether a respondent was interviewed before or after the outbreak of the pandemic (COVID dummy), we include several independent variables to uncover to what extent the crisis had a differential impact on different social groups and in different contexts. First, we use subjective income, which has a lower number of missing values than objective income measurements. Respondents were asked which of the following four categories best describes their total household income: ‘We have more than enough, we can easily save’, ‘We have enough and have no difficulties getting by’, ‘We have just enough to get by’ and ‘We do not have enough and regularly have difficulties getting by’. We created a dummy that merges the first two into one category and the last two into another (0 = high income; 1 = low income). It should nevertheless be noted that models using objective operationalizations instead of subjective measurements of income yielded similar results (see also Choi 2021 for similar impacts of objective and subjective income on support for a BI). Second, group-relative deprivation is operationalized by three five-point agree/disagree statements. In particular, the items examine whether individuals feel that their group ‘always has to wait longer than others’, that they ‘are systematically disadvantaged’ and that they ‘are always the first victims of an economic crisis’. A Confirmatory Factor Analysis (CFA) indicates that these three items all load strongly on an underlying latent concept (see Table A3 in the Online Appendix). Factor scores for this latent concept, as well as for its interaction with the COVID dummy, are saved so that they can be included in our models. Third, to determine whether the crisis had a different effect across regional contexts, a dummy variable is included that indicates whether respondents live in Flanders or in Francophone Belgium (0 = Flanders; 1 = Francophone Belgium). As no Dutch-speaking individuals were interviewed in Brussels, all respondents living in Brussels (*N* = 102) are categorized as belonging to Francophone Belgium, which also includes all respondents living in Wallonia. Fourth, to test the persistence of a decrease or increase in support of a BI, we include the number of COVID-19 related hospitalizations as an independent variable. More specifically, this indicator measures the number of hospitalizations at the time the respondents were interviewed, which varies across time and, thus, across respondents.

Last, we include a number of variables in our model to control for confounding factors. In addition to age and gender (0 = female; 1 = male), two dummies for educational levels are used (none to lower secondary; upper secondary = reference category; tertiary). Left–right political placement is also included, which is assessed on an 11-point scale (0 = left; 10 = right). To control for the mode of interviewing, three dummies are added (CAWI; video calls; CAPI = reference category).

### Modelling strategy

To answer our first research question and see how support for a BI evolved after the start of the crisis, we first provide a descriptive overview that visualizes the overall means of support for a BI (averaged across all vignettes) over time. To assess the likely persistence of any shift in public support for a BI, we also test here to what extent such support fluctuated with the number of hospitalizations due to COVID-19, which we include as an independent variable in our regression models (for the COVID sample only). The underlying assumption is that if the opinion shift is stable in the long term, we would not expect to see large short-term fluctuations driven by factors that will cease to exist once the pandemic has come to an end (such as COVID-induced hospitalizations). Importantly, the number of hospitalizations was used by the Belgian government as *the* yardstick to assess the severity of the health crisis and to introduce or maintain a wide range of COVID-19 related measures, including lockdowns. This implies that the number of hospitalizations can also serve as a proxy for the severity of the economic crisis. Indeed, a greater number of hospitalizations meant that stricter measures were taken, and these were usually associated with economic decline. To investigate the extent to which the shift in opinion is actually BI specific, we make use of the internal variation present in our vignette experiment. Accordingly, we calculate the difference between the Average Marginal Component Effect (AMCE) of support for different types of BI in the COVID and pre-COVID samples, by estimating a regression model with interactions between the vignette dimensions and the COVID dummy. By assessing the differences in AMCEs between the COVID sample and pre-COVID sample, we are able to assess which types of BI became more or less popular, relative to the ideal–typical form.

To answer our second research question, we estimate two stepwise regression models to assess the direct impact of the crisis. While the first model includes the COVID dummy only, the second model adds the interaction terms between the COVID dummy and the indicators of deprivation. To control for the differences in the composition of both samples, these regression models also include all of the other independent variables, thereby making the COVID and pre-COVID samples as comparable as possible.

## Results

### The evolution of support for a BI

**Fig. 1:**
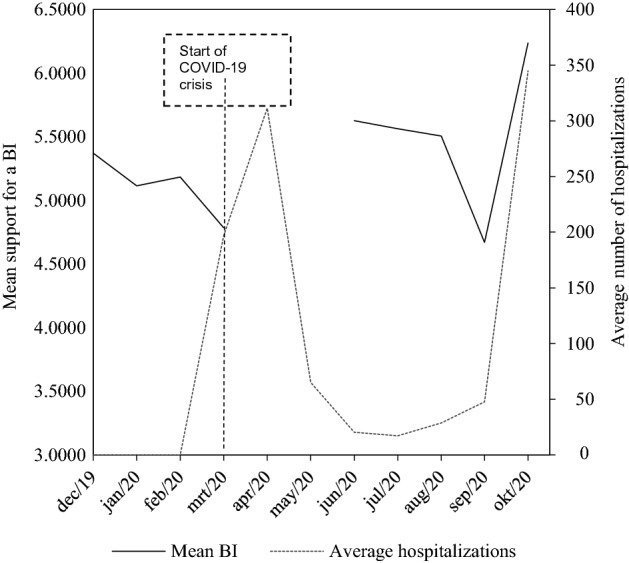
Evolution of public support for a BI and the number of COVID-19 hospitalizations over the months of data collection. Data collection lasted up to November 2020, but only 16 respondents were interviewed in that month so they are excluded from this graph

In Fig. 1, we visualize mean support for a BI over the months of data collection as a first indication of how it evolved after the outbreak of the pandemic. The average number of hospitalizations over the seven days prior to the survey is also visualized to see the extent to which support for a BI runs parallel to increased risk exposure. While the number of hospitalizations is a direct indicator of the intensity of health risks, it also offers a proxy of economic and social risks, as higher numbers of hospitalizations coincided with stricter COVID-19 regulations that had a massive impact on economic and social life. It should be noted that almost all the respondents in March 2020 were interviewed before the start of the pandemic, and that no interviews were conducted in April and May 2020, thereby leading to missing values for the mean support for a BI.

First, Fig. 1 indicates that before the outbreak of the virus, support for a BI was relatively stable, fluctuating between 5.37 and 4.78 on the 0–10 answer scale. However, between March 2020 and June 2020, support for a BI increased more strongly, which is presumably attributable to the start of the COVID-19 crisis. We also see that there were stronger fluctuations after the start of the pandemic, as support remained relatively stable during the summer, declined in September and rose to a level of mean support of 6.24 in October. To some extent, support for a BI seems to run parallel to the number of hospitalizations, as the stability in support during the summer months coincided with a relatively stable number of hospitalizations, and the major increase in support in October corresponded to the start of the second wave of the pandemic in Belgium. To test this in greater detail, we also use a regression model to explicitly look at the impact of the number of hospitalizations on support for a BI (For a more detailed description of the methodology, see the Online Appendix). Figure A1 in the Online Appendix shows that the number of hospitalizations did indeed have a significant positive effect on support for a BI. Similar to Fig. 1, there are important fluctuations within the COVID-19 sample that appear to follow the number of hospitalizations, which points to an important short-term effect of the crisis. Furthermore, this finding strengthens the conclusion that the increased risk exposure in light of the COVID-19 crisis was responsible for an increase in support for a BI. This finding also suggests, however that the increase in support is unlikely to persist after the pandemic has ended, as the number of COVID-induced hospitalizations will come to an end at some point.

As mentioned in the theory section, the goal is also to check which types of BI became more (or less) popular, and, thus, to assess which attributes of a BI became more (or less) attractive. To test this, we calculate the difference in AMCEs of the different attributes in the two samples. These differences are equal to the interaction effects between the vignette dimensions and the COVID-19 dummy variable. These values only express relative differences in the popularity of different designs across samples, and do not reveal which types of BI have most support in absolute terms. This is visualized in Figure A2 in the Online Appendix. In contrast to what could intuitively be expected, the proposals that deviate somewhat from the ideal–typical BI were the ones that gained relative popularity in times of the pandemic, relative to the start of it. In comparison to a fully universal BI, a proposal that excludes the rich, for instance, became somewhat more attractive. Similarly, a BI that grants higher amounts to those in need or to those who have worked longer, and schemes that are conditional in nature seem to gain support compared with egalitarian and unconditional proposals. Although somewhat surprising, this is in line with previous research suggesting that a BI can also be attractive from a selectivist point of view (Lee 2021). Nevertheless, the largest (and only statistically significant) difference is observed for the integration dimension, where a proposal that replaces all existing benefits, including pensions, is significantly more popular. While it is surprising that the increase in relative support is especially noticeable when pensions (rather than unemployment benefits) are mentioned, the rising popularity of a BI that replaces existing schemes compared to the non-replacing BI could point to the increasing appeal of simplicity and efficiency in welfare provision during the COVID-19 crisis (Nettle et al. 2021). Last, for the accumulation dimension, we see that proposals that do not allow accumulation become slightly, albeit not significantly, less popular compared with the ideal–typical BI that enables accumulation of earnings. Overall, these results indicate that increase in support for a BI does not necessarily reflect an increase in support for the universal, unconditional ideal–typical version. Accordingly, the case could be made that the observed shift in support after the onset of COVID-19 may perhaps not be specific to a BI, but instead relates to welfare provision more generally.

### The impact of the COVID-19 crisis on support for a BI across groups with varying levels of deprivation

We estimate two regression models to assess the extent to which the COVID-19 crisis affected support for a BI overall, and for groups with different levels of deprivation specifically. Table 1 shows the unstandardized coefficients for a model including only the COVID-19 dummy and the control variables (for a discussion of the impact of the control variables, see the Online Appendix), as well as for a second model including the interactions with the individual, group-based and context-related indicators of deprivation. The first model is still useful to estimate on top of the descriptive overview, as it controls for the different sample compositions before and after the outbreak of the pandemic.

**Table 1:** Impact of the COVID-19 crisis and its interactions with deprivation on public support for a BI (*N* = 1515)

	Model 1	Model 2
Variables of main interest		
COVID-19		
No (ref.)		
Yes	0.780***	0.229
Subjective income		
More than enough or no difficulties (ref.)		
Just sufficient or difficulties	0.147	0.221
Region		
Flanders (ref.)		
Francophone Belgium	0.125	− 0.306
Group-relative deprivation	0.159	− 0.068
Subjective income × COVID		− 0.223
Francophone Belgium × COVID		0.965**
Group-relative deprivation × COVID		0.466*
Control variables: vignette dimensions		
Universality		
Fully universal (ref.)		
Universal based on residency	− 0.049	− 0.046
Selective: excluding rich	0.354	0.366
Selective: including the poor	0.187	0.212
Uniformity		
Equality (ref.)		
Need	0.058	0.045
Equity	0.390*	0.407*
Conditionality		
Unconditional (ref.)		
Conditional on job seeking	0.560***	0.562***
Conditional on participation	0.476**	0.480**
Integration		
No replacement (ref.)		
Replacement pension	0.142	0.150
Replacement unemployment	0.169	0.171
Accumulation		
Accumulation (ref.)		
No accumulation	− 0.183	− 0.180
Control variables: Individual characteristics		
Gender		
Female (ref.)		
Male	− 0.107	− 0.106
Age	− 0.002	− 0.001
Education level		
None to lower secondary	0.305	0.289
Upper secondary (ref.)		
Tertiary	0.178	0.120
Survey mode		
CAPI (ref.)		
CAWI	− 0.787***	− 0.460
Video call	− 0.066	0.340
Left–right placement	− 0.165***	− 0.158***
*R*^2^	0.083	0.094

***p ≤ 0.001; **p ≤ 0.010; *p ≤ 0.050

**Fig. 2:**
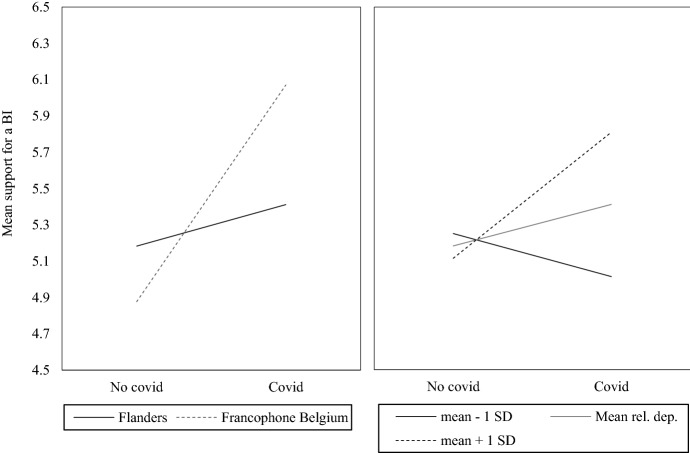
Visualization of interaction terms. SD refers to the standard deviation, as relative deprivation is saved as factor scores

The first model displayed in Table 1 shows that the COVID-19 crisis led to a significant increase in support for a BI. In line with the government protection hypothesis (H1a), support for a BI increased in response to the heightened risk exposure during the pandemic. However, the coefficient for the effect of the COVID-19 crisis becomes insignificant in the second model, where we added interactions between the COVID dummy and our indicators for deprivation. The fact that two of these interactions are significant might explain why the main effect of the COVID-19 crisis disappears. While no interaction is found with the subjective income variable, the interactions for group-relative deprivation and the regional context are significant. This shows that differences in the impact of the pandemic occur more strongly when deprivation is group or context related. It should be noted that even when testing alternative models including a different indicator of individual deprivation, such as education level or objective income instead of subjective income, the interactions do not change. In each of these models, the individual indicator of deprivation is not significant, while for group-relative deprivation and regional context the interactions are significant. To gain more insight into these interactions, they are visualized in Fig. 2.

Figure 2 shows that the COVID-19 crisis had a very different impact in Flanders than in Francophone Belgium. While in Flanders we see a relatively strong degree of stability, in Francophone Belgium there was a substantial increase in support for a BI after the start of the crisis. In particular, support for a BI increased by more than one point on an eleven-point scale in Francophone Belgium. Material and social deprivation is still much greater in Francophone Belgium than in Flanders (Working Group Social Impact Corona Crisis 2021), which may explain why citizens living in these regions reacted so differently in terms of their support for the introduction of a BI.

Figure 2 also indicates that similar to the regional context, the COVID-19 crisis affected support for a BI quite differently across groups with varying levels of relative deprivation. As the latent concept of group-relative deprivation was converted into factor scores, the different lines of the graph represent groups that score one standard deviation below the mean, groups that have a mean score and groups that score a standard deviation above the mean. For those with a mean score on group-relative deprivation, support for a BI is fairly similar before and after the outbreak of the pandemic. However, for groups that experience a strong sense of relative deprivation, the COVID-19 crisis led to more positive inclinations towards the implementation of a BI. For those with lower than average deprivation, the crisis had exactly the opposite effect, as their levels of support for a BI dropped slightly. Overall, these interactions are in contrast to the parallel publics’ hypothesis (H2b) and instead seem to lend support to the polarization hypothesis (H2a), which states that groups respond very differently to an increase in crisis-induced risk exposure. Consequently, the government protection thesis (H1a) does not seem to hold universally, but applies more specifically to deprived groups and contexts.

## Conclusion and discussion

The COVID-19 crisis has shaken societies around the world to their foundations. The question that arises is whether these profound changes also fundamentally altered public opinion. To provide more insight into these pressing issues, the current study evaluated whether support for a BI changed in response to the pandemic, and whether this differed across social groups and contexts with varying levels of deprivation. Intuitively, one might expect an increase in support for a BI, as a wave of solidarity seems to have resonated across European societies. However, people might be equally likely to become less altruistic, and changes in opinions might diverge for various societal groups. Consequently, this is the first study to empirically assess changes in public support for a BI in light of the COVID-19 crisis by using a natural experiment to compare responses before the start of the pandemic with those of respondents interviewed in the midst of the crisis.

Based on data from the 2019–2020 Belgian National Elections Study, the results indicate that support for a BI has indeed increased overall since the outbreak of the pandemic. In line with the government protection thesis, greater risk exposure seems to heighten the demand for redistribution and government protection (Blekesaune 2007; Jæger 2013; Sachweh 2018), which in turn translates into more support for a BI. However, our results equally point towards a number of important nuances with regard to this trend, as it is not necessarily (1) universal, (2) long lived and (3) fully BI specific. First, we demonstrate that the shift in support for a BI is only observable for groups that feel relatively deprived and for respondents living in the more deprived region of Francophone Belgium. In line with the polarization thesis (Brito Vieira et al. 2017; Forma 2002), the intensification of risk exposure has a different effect on various groups. The government protection hypothesis, thus, does not seem to apply universally, but is specific to those who are already deprived. Second, we demonstrate that fluctuations in support for a BI appear to follow the number of hospitalizations due to the pandemic. This finding could indicate that the increase in support for a BI is not long term, but is instead driven by upsurges in economic, social and health-related insecurities that are likely to fade away in the post COVID-19 era. Last, the results show that it is not necessarily support for a universal and unconditional type of BI that has gained popularity. We show that more-restrictive types of BI (e.g. selective and conditional) and especially a BI that replaces existing benefit schemes (pensions in particular) gained support relative to the unconditional and universal BI. Consequently, we conclude that the observed increase in support for a BI may well reflect an increase in support for welfare provision in general.

Despite being the first to offer a nuanced, detailed and innovative empirical perspective on the evolution of support for a BI since the start of the COVID-19 crisis, this study contends with shortcomings that should be resolved in future research. First, our data only run up to November 2020, hence, making it difficult to draw conclusions about the long-term impact of the crisis on support for a BI. Future research would, thus, benefit from following up on these issues and determining whether support returns to pre-crisis levels after the pandemic has ended. Second, our approach does not allow us to rule out other external factors—such as increased political salience or media framings—that occurred simultaneously with the development of the pandemic and might have influenced support for a BI. However, given that the crisis dominated the news and politics, even these changes in framing would probably especially occur in relation to the COVID-19 crisis. Third, our focus was particularly on a BI, as it is seen as an attractive scheme to solve some of the challenges arising from the pandemic. However, additional research should be done on how opinions about a BI compare with attitudes towards other types of social policies. The current study nevertheless opens up the way for these studies by illustrating that the COVID-19 crisis did have an impact on public support for a BI, but that simultaneously nothing is as simple as it seems.

## Supplementary Information

Below is the link to the electronic supplementary material.

Supplementary file1 (DOCX 35 kb)
