# Supplementary material for: A wave of support? A natural experiment on how the COVID-19 pandemic affected the popularity of a basic income
Source: Acta Polit. 2022 Oct 5;58(3):695–713. doi: 10.1057/s41269-022-00260-9 (PMC9533981; doi:10.1057/s41269-022-00260-9)
Supplement: Supplementary file 1 — Supplementary file1 (DOCX 35 kb) [file 41269_2022_260_MOESM1_ESM.docx]

**Online Appendix**

**Explanation impact of the number of hospitalizations**

To evaluate whether the opinion shift is persistent or driven by short-term risk exposure, we look at the role of the number of hospitalizations in shaping public opinion on a BI. To assess this, the seven-day average of the number of daily admissions to hospitals is assigned to an individual on the basis of their date of interview. If there have for instance been 100 hospital admissions on average over the last seven days at the moment when the respondent is interviewed, the respondent is assigned a score of 100 on this variable. We specifically look at the seven-day average of the hospitalizations, as this is less sensitive to outliers and does not rely on the testing strategy to map the number of Covid-19 infections. These data are taken from Sciensano, which is the Belgian institute responsible for the epidemiological follow-up of the Covid-19 epidemic. Although the number of hospitalizations is a crude proxy to measure the economic impact of the pandemic and mainly refers to exposure to health risks, it is one of the only indicators that varies sufficiently on a daily basis to use it as an individual-level variable. Furthermore, this indicator still offers a rather all-round measurement of the severity of the crisis that extends to economic (e.g., repercussions lockdowns) and social (e.g., intensity social contacts) domains as well. To test the influence of the hospitalizations, a regression model is applied only on the Covid-sample, wherein all of the other independent variables are also included. To consider that individuals who were interviewed in subsequent days are ascribed equivalent numbers of hospitalizations and hence to account for the partial non-independence of observations, robust standard errors are estimated

**Discussion of the impact of the control variables**

Besides the main relationships of interest, Table 1 in the main text displays the regression coefficients of the control variables. Although most variables do not have a significant influence, we see that two of the design characteristics of a BI have a substantial impact: schemes that are conditional on job-seeking or volunteering as well as proposals that are more generous towards those who have worked longer receive significantly more support than their unconditional and egalitarian counterparts. In addition, in line with previous research, left-right placement relates significantly to support for a BI, whereby more right-wing respondents are less in favour of a BI (Roosma and van Oorschot 2019). Last, while the survey mode does have a significant influence in the first model, this disappears completely in the second model. That respondents who used the web-based survey score lower on support for a BI, might be related to the weaker differentiation in rating scales among these respondents, whereby a larger share opts for the middle point of the scale (Heerwegh and Loosveldt 2008).

**Supplementary Tables**

Table A1. Descriptive statistics and logistic regression of membership for the pre-Covid and Covid sample (cf. Jensen & Naumann, 2016)

|  | **Pre-Covid**  **(control group)** | **Covid**  **(treatment group)** | **Odds ratio in treatment group** |
| --- | --- | --- | --- |
| **Support basic income** | 5.12 | 5.66 |  |
| **Gender** |  |  |  |
| Female (ref.) | 50.50 % | 51.70 % |  |
| Male | 49.50 % | 48.30 % | 1.01 |
| **Age** | 50.26 | 47.38 | 0.99*** |
| **Education level** |  |  |  |
| No to lower secondary | 26.10 % | 30.80 % | 1.531** |
| Higher secondary (ref.) | 39.70 % | 34.90 % |  |
| Tertiary | 34.20 % | 34.30 % | 1.182 |
| **Left-right placement** | 5.07 | 4.77 | 1.01 |
| **Subjective income** |  |  |  |
| More than enough or no difficulties (ref.) | 68.60 % | 64.60 % |  |
| Just sufficient or difficulties | 31.40 % | 35.40 % | 0.78 |
| **Region** |  |  |  |
| Flanders (ref.) | 80.30 % | 32.40 % |  |
| Francophone Belgium | 19.70 % | 67.60 % | 9.11*** |
| **Group relative deprivation** | 3.01 | 3.21 | 1.11 |

Table A2. Dimensions, levels and vignette text for the basic income proposals

| **Dimension** | **Level** | **Vignette text** |
| --- | --- | --- |
| **Universality** | Fully universal | *The Belgian government pays a monthly income to all residents* |
|  | Universal based on residency | *The Belgian government pays a monthly income to all residents, if they have lived a couple of years in our country* |
|  | Selective: excluding the rich | *The Belgian government pays a monthly income to all residents, except to those who have a lot of money* |
|  | Selective: only the poor | *The Belgian government pays a monthly income only to residents who have little money* |
| **Uniformity** | Equality | *The amount is equal for everyone* |
|  | Need | *The amount is lower as people earn more* |
|  | Equity | *The amount is higher as people have worked longer* |
| **Conditionality** | Unconditional | *People who are not working are not obliged to search for a paid job* |
|  | Conditional on job-seeking | *People who are not working are obliged to search for a paid job* |
|  | Conditional on participation | *People who are not working are obliged to do voluntary work or to take up caring responsibilities* |
| **Integration** | No replacement | *The basic income replaces no other social benefits* |
|  | Replacement pension | *The basic income replaces all existing social benefits, such as pensions* |
|  | Replacement unemployment | *The basic income replaces all existing social benefits, such as unemployment benefits* |
| **Accumulation** | Accumulation | *People receive the basic income on top of the money they earn by working or in another way* |
|  | No accumulation | *The basic income is diminished with the money people earn by working or in another way* |

Table A3. Factor loadings and question wordings for group relative deprivation

|  | **Group relative deprivation** |
| --- | --- |
| Item 1 - If we need something from the government, people like me always have to wait longer than others | 0.799 |
| Item 2 - People like me are systematically disadvantaged, while other groups receive more than they are entitled to | 0.853 |
| Item 3 - People like me are always the first victims of an economic crisis | 0.771 |

**Supplementary Figures**

b = 0.001***

Figure A1. Relationship between number of hospitalizations and mean support for a BI (under control of all variables included in previous regression models; N = 639).

Figure A2. Differences in AMCEs for the vignette dimensions between the pre-Covid and Covid sample
